# Statistical Process Control Charts for Monitoring Next-Generation Sequencing and Bioinformatics Turnaround in Precision Medicine Initiatives

**DOI:** 10.3389/fonc.2021.736265

**Published:** 2021-09-24

**Authors:** Sneha Rajiv Jain, Wilson Sim, Cheng Han Ng, Yip Han Chin, Wen Hui Lim, Nicholas L. Syn, Nur Haidah Bte Ahmad Kamal, Mehek Gupta, Valerie Heong, Xiao Wen Lee, Nur Sabrina Sapari, Xue Qing Koh, Zul Fazreen Adam Isa, Lucius Ho, Caitlin O’Hara, Arvindh Ulagapan, Shi Yu Gu, Kashyap Shroff, Rei Chern Weng, Joey S. Y. Lim, Diana Lim, Brendan Pang, Lai Kuan Ng, Andrea Wong, Ross Andrew Soo, Wei Peng Yong, Cheng Ean Chee, Soo-Chin Lee, Boon-Cher Goh, Richie Soong, David S.P. Tan

**Affiliations:** ^1^ Yong Loo Lin School of Medicine, National University of Singapore, Singapore, Singapore; ^2^ Department of Haematology-Oncology, National University Cancer Institute, Singapore, Singapore; ^3^ Cancer Science Institute of Singapore, National University of Singapore, Singapore, Singapore; ^4^ Department of Pathology, Yong Loo Lin School of Medicine, National University Health System, Singapore, Singapore; ^5^ Department of Pathology, National University Hospital, National University Health System, Singapore, Singapore; ^6^ Department of Pharmacology, Yong Loo Lin School of Medicine, National University Health System, Singapore, Singapore; ^7^ Department of Medicine, Yong Loo Lin School of Medicine, National University Health System, Singapore, Singapore; ^8^ Pascific Laboratories, Singapore, Singapore

**Keywords:** precision medicine, computational biology, next generation sequencing, precision oncology, bioinformatics

## Abstract

**Purpose:**

Precision oncology, such as next generation sequencing (NGS) molecular analysis and bioinformatics are used to guide targeted therapies. The laboratory turnaround time (TAT) is a key performance indicator of laboratory performance. This study aims to formally apply statistical process control (SPC) methods such as CUSUM and EWMA to a precision medicine programme to analyze the learning curves of NGS and bioinformatics processes.

**Patients and Methods:**

Trends in NGS and bioinformatics TAT were analyzed using simple regression models with TAT as the dependent variable and chronologically-ordered case number as the independent variable. The M-estimator “robust” regression and negative binomial regression were chosen to serve as sensitivity analyses to each other. Next, two popular statistical process control (SPC) approaches which are CUSUM and EWMA were utilized and the CUSUM log-likelihood ratio (LLR) charts were also generated. All statistical analyses were done in Stata version 16.0 (StataCorp), and nominal P < 0.05 was considered to be statistically significant.

**Results:**

A total of 365 patients underwent successful molecular profiling. Both the robust linear model and negative binomial model showed statistically significant reductions in TAT with accumulating experience. The EWMA and CUSUM charts of overall TAT largely corresponded except that the EWMA chart consistently decreased while the CUSUM analyses indicated improvement only after a nadir at the 82^nd^ case. CUSUM analysis found that the bioinformatics team took a lower number of cases (54 cases) to overcome the learning curve compared to the NGS team (85 cases).

**Conclusion:**

As NGS and bioinformatics lead precision oncology into the forefront of cancer management, characterizing the TAT of NGS and bioinformatics processes improves the timeliness of data output by potentially spotlighting problems early for rectification, thereby improving care delivery.

## Introduction

Precision oncology refers to the use of therapeutics expected to benefit patients who harbor specific molecular or histopathological biomarkers (1). Due to the known complexity of cancers and the expanding body of knowledge of oncogenesis, molecular profiling, such as next generation sequencing (NGS) molecular analysis and bioinformatics, is being used to identify genetic mutations (2, 3), which may guide the deployment of targeted therapies (4, 5). As precision oncology remains a cornerstone in treating cancer patients and has been proven to improve outcomes (6, 7), its application is likely to be imperative in cancer research, clinical practice and patient care in the years to come. However, a critical element to this enterprise is the laboratory turnaround time (TAT), which refers to the time from sample receipt to the return of molecular results (8).

The Integrated Molecular Analysis of Cancers (IMAC) study was conducted to establish the prevalence and range of mutations in Asian patients with advanced cancers (9) to further develop and understand the value of targeted molecular profiling and gene sequencing in clinical decision making. TAT is a key performance indicator of laboratory performance (8) and a shorter TAT indicates that actionable information is being provided in a timely fashion (10), which expedites the decision-making process in management and treatment. Furthermore, a shorter TAT allows for the prudent use of resources in public health as patients can be discharged more quickly and require less hospital in-patient services (11). However, NGS labs are often hampered by various logistical and technical difficulties (12) and bioinformatics challenges (13).

However, the quantification of the learning curves for next generation sequencing labs has yet to been described in literature. As such, a first step in measuring the real-time efficiency and identifying bottlenecks in NGS and bioinformatics pipelines could be to adopt statistical process control (SPC) methods such as cumulative sum (CUSUM) (14), or exponentially weighted moving average (EWMA) (15), to monitor the temporal parameter stability and identify structural breakpoints in TAT. CUSUM is a sequential analysis technique which monitors change detection, particularly deviation from a performance standard (16–18), and has been widely used in a range of healthcare settings, from describing the learning curves of surgical or procedural skills (19–24), to clinical audits (25–27), and quality-assurance studies (22, 28–32). Similar to CUSUM, EWMA control charts have been widely used for measuring and monitoring healthcare outcomes (33, 34). EWMA analysis has been shown to detect smaller shift sizes that occur especially during the earlier period of monitoring (35).

To our knowledge, this study is the first to formally apply statistical process control (SPC) methods such as CUSUM and EWMA to a precision medicine programme to characterize the turnaround time for molecular report generation and analyze the learning curve of NGS and bioinformatics in the precision medicine era.

## Methods

### Patients

The characteristics and eligibility criteria of patients recruited to the Integrated Molecular Analysis of Cancers (IMAC) precision medicine platform has been described previously (9). Briefly, patients referred to the Developmental Therapeutics Unit (DTU) at the National University Cancer Institute, Singapore (NCIS) were offered participation in the IMAC programme if they were above 21 years of age, and had a histologically confirmed diagnosis of a solid malignancy or lymphoma, and had adequate tumor tissue for genome characterization. The IMAC protocol was approved by the National Healthcare Group-Domain Specific Review Board (NHG-DSRB) and the SingHealth Centralized Institutional Review Board and was undertaken in adherence with the Good Clinical Practice Guidelines. Written and informed consent was obtained from patients before study entry. Genomic profiles were characterized from a variety of pathological specimens (including tissue block or frozen specimens, slides, or cytological samples such as smears, and CT-guided or EUS guided, biopsy-imprint or FNA smear cytology, and DNA) retrieved during routine clinical procedures (e.g., surgery, biopsy, or ascites and pleural fluid drain).

### Sequencing and Bioinformatics Analyses

As described previously (9), targeted next-generation sequencing was performed using the AmpliSeq Cancer Hotspot Panel v2 on an Ion Torrent/PGM system (Life Technologies, CA, USA) per the manufacturer’s instructions. We rejected samples with an average sequencing depth <500, unfiltered DNA variants >100, or uniformity of coverage <80% from bioinformatics analyses. The pathogenicity of exonic non-synonymous alterations were individually reported after reviewing published literature and public curated databases (i.e., ClinVar for pathogenicity; My Cancer Genome for variant frequency and exon number; Ensembl GRCh37 for the protein domain). When phenotypic information on rare variants were missing from ClinVar or Catalogue of Somatic Mutations in Cancer (COSMIC) databases, we interrogated Variant Call Format (VCF) files with Variant Effect Predictor (VEP) version 75 to infer their *in silico* pathogenicity (SIFT and POLYPHEN). As we did not perform paired normal tissue profiling, we excluded variants with minor allele frequency >5% among South East Asians in the 1000 Genomes Database. Since the evidence base for precision oncology biomarkers was in constant flux, we did not utilize any predetermined protocols to prioritize the actionability of reported variants.

### Learning Curve Analyses

We began analyses of trends in TAT using simple regression models in which the dependent variable was the TAT and the independent variable was the chronologically-ordered case number. Two modelling approaches were chosen and serve as sensitivity analyses to each other: (i) M-estimator “robust” regression, in which a biweight loss function is minimized through an iteratively-weighted least squares algorithm, thus minimizing the impact of influential outliers; and (ii) negative binomial regression, since the turnaround time (in days) could constitute a form of ‘count data’ and therefore may be assumed to follow a Poisson-related family distribution.

Next, to illustrate the application of statistical process control (SPC) to the precision medicine paradigm, we exploited two popular SPC approaches: CUSUM and EWMA. We did not use risk-adjusted models as no predictors of TAT could be identified despite using multivariable linear, quantile, or negative binomial models, and evaluating a range of potential predictors (e.g., type of specimen [tissue block vs other types of specimens such as pap smears or fine needle aspiration], tumor content of specimen, etc.). The CUSUM statistic was calculated as the running sum of residuals (expected minus observed [O_j_] outcome values) over chronologically-ordered patients, as per the following equation:


$$C_{j}=\sum_{k=1}^{j}\left(E_{j}-O_{j}\right)$$

in which the expected value (Ej) refers to the mean TAT. On the basis of this equation, downward trends indicate that the observed values exceed the expected value (i.e., turnaround time for specimen greater than expected), whereas upward trends indicate an improvement in performance.

CUSUM log-likelihood ratio (LLR) charts were also generated, using clinically-selected thresholds of >6 weeks for overall TAT, and >3 weeks each for the wet-lab and dry-lab components of NGS respectively. To identify clinically-meaningful signals, we chose an odds ratios of 1.20 (e.g., a 20% increase in the odds of overall turnaround time > 42 days) and 1/1.20 = 0.83 (i.e. a 17% decrease in the odds of overall turnaround time > 42 days) as the alternative hypotheses. However, the CUSUM chart was not reset when the control limits were exceeded. The exponentially weighted moving average (EWMA) chart has been proposed to provide an ongoing local estimate of the average score that is purportedly easier for clinical staff to interpret and understand (19, 20). As such, we also illustrated the application of EWMA control chart for monitoring trends in TAT. The mean of the first ten observations were used to obtain the initial value for recursion, and the EWMA was calculated by assigning lesser weight to observations earlier in the series in a geometrically-declining fashion (decay factor: 0.1), with a smoothing parameter of alpha = 0.03. All statistical analyses were done in Stata version 16.0 (StataCorp), and nominal P < 0.05 were regarded to indicate statistical significance. All scripts were uploaded at the Github website (https://github.com/nicholassyn/Stata-codes-for-IMAC-CUSUM-and-EWMA-analysis/tree/main).

## Results

### Patient Information

A total of 365 patients with a variety of advanced malignancies underwent successful molecular profiling using the AmpliSeq Cancer Hotspot Panel v2 as part of the IMAC molecular screening initiative (9). The molecular landscape and histopathological characteristics of these patients have been reported previously (9).

### Overall Learning Curve

**Figure 1:**
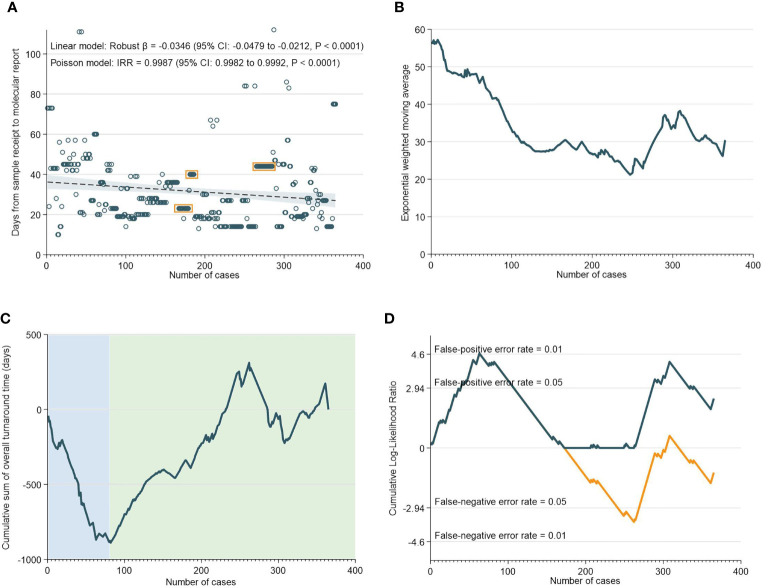
Overall Turnaround Time. **(A)** Scatter and regression fit of overall turnaround *vs* case load. **(B)** Exponential weighted moving average of overall turnaround time. **(C)** CUSUM of overall turnaround time. **(D)** CUSUM log-likelihood ratio chart for overall turnaround time > 3 weeks.

Overall, there was a median of 27 days (IQR: 19-43) between the receipt of the sample to the generation of the molecular report. As indicated in **Figure 1A**, both the robust linear model (Robust β= -0.0346; 95% CI: -0.0479 to -0.0212, p<0.0001) and negative binomial model (IRR=0.9987; 95% CI: 0.9982 to 0.9992, p<0.0001) showed statistically significant reductions in TAT with accumulating experience. The EWMA chart of overall TAT (**Figure 1B**) showed that TAT consistently decreased from 0 to 365 cases, although interjected by a transient increase in TAT between 250 to 310 cases. CUSUM analyses of overall TAT indicated that from 0 to 82 cases, there was a worsening trend and the chart reached its nadir at 82. Subsequently, the CUSUM chart showed an improving trend from 82 to 365 cases with the exception of a deteriorating trend from 265 to 310 cases. The deteriorating phase corresponds largely with the findings in the EWMA chart which showed an increase in TAT from 250 to 300 cases (**Figures 1C, D**).

### Learning Curve of NGS Assays (Wet-Lab Component)

**Figure 2:**
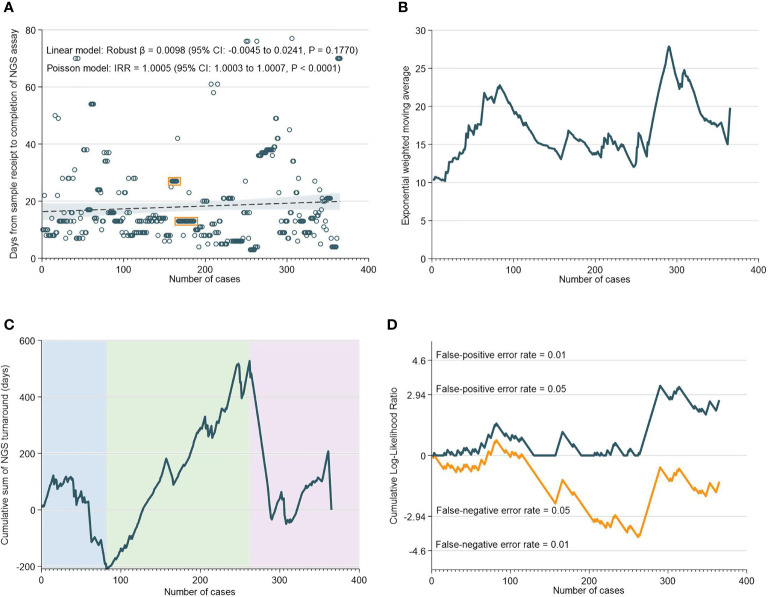
Turnaround Time for NGS Assays. **(A)** Scatter and regression fit of NGS turnaround *vs* case load. **(B)** Exponential weighted moving average of NGS turnaround time. **(C)** CUSUM of NGS turnaround time. **(D)** CUSUM log-likelihood ratio chart for nGS turaround time > 3 weeks.

There was a median of 13 days (IQR: 9-21) from the receipt of the sample to the NGS assay result. Interestingly, robust regression of NGS assay TAT versus case numbers did not reveal any statistically-significant temporal trend; in fact, negative binomial regression unexpectedly indicated a possible increase in TAT over time (**Figure 2A**). EWMA analyses also indicated that the moving average TAT estimate was higher at almost all timepoints as compared to the initial ~20 cases (**Figure 2B**). CUSUM analyses corroborated these findings, and demarcated three time periods: an initial increase in TAT up to 85 cases (nadir at 82 cases), followed by a decrease in TAT up to 265 cases, and then a subsequent increase in TAT up till the 365th case. Notably, the increase in NGS turnaround time at the 262nd case approximately corresponds with a period of transition and changes in key lab personnel (**Figures 2C, D**).

### Learning Curve of Bioinformatics Analyses (Dry-Lab Component)

**Figure 3:**
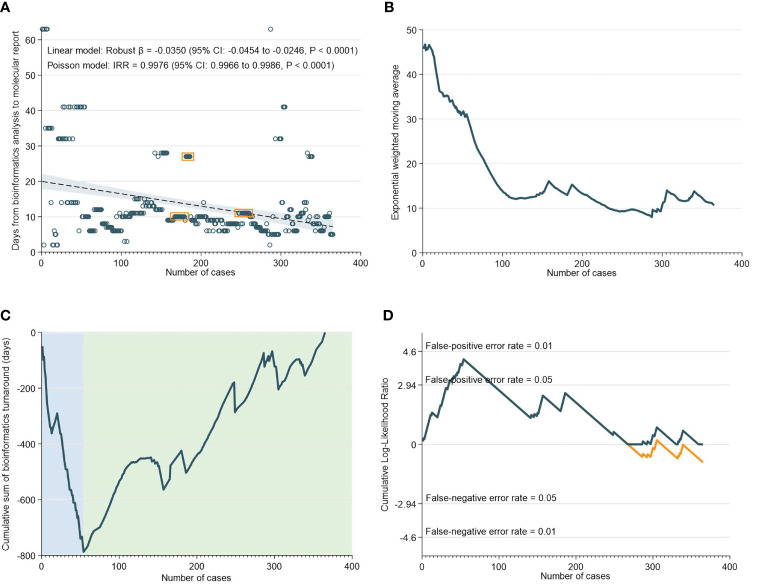
Turnaround Time for Bioinformatics Analyses. **(A)** Scatter and regression fit of bioinformatics turnaround *vs* case load. **(B)** Exponential weighted moving average of bioinformatics turnaround. **(C)** CUSUM of bioinformatics turnaround time. **(D)** CUSUM log-likelihood ratio chart for bioinformatics turnaround > 3 weeks.

There was a median of 10 days (IQR: 8-13) from NGS result to generation of the bioinformatics report. As demonstrated in **Figure 3A**, the TAT for bioinformatics analyses decreased significantly over time. This was supported by the EMWA analysis, which shows a rapid decrease in TAT up to the 110th case, followed by a more gradual pace of decrease subsequently (**Figure 3B**). The CUSUM analyses, however, revealed that between the 0th to 54th case, there was an initial increase in TAT, only after which did the turnaround time for bioinformatics analyses begin to improve (**Figures 3C, D**).

## Discussion

Learning curve analysis has been widely used in surgery (29, 36–41), due to its usefulness in presenting the performance of surgeons using various outcome measures relative to their cumulative case loads (18, 42–44). This enables the objective quantification of caseloads required for surgeons to surmount their learning curves and spotlights performance deviation that necessitates intervention (18, 36, 45). In oncology, precision medicine has been shown to achieve improved therapeutic outcomes and is becoming increasingly prevalent (46). NGS allows for sequencing of the whole genome to identify molecular aberrations instead of a singular biomarker, coupled with bioinformatics, targeted approaches could be identified to treat cancers and advance the effectiveness of personalized medicine in the years to come (47). However, the complex nature of NGS and bioinformatics are barriers to their ubiquitous use due to logistical and technical difficulties (9). In order to improve lab performances, the characterization of learning curve in NGS and bioinformatics could prove useful in facilitating greater adoption of precision oncology and accelerate the move towards comprehensive genomic profiling (48). To our knowledge, this is the first paper to characterize the learning curves of both NGS and bioinformatics, two key components in precision oncology.

The RA-CUSUM analysis found that the bioinformatics team took a lower number of cases (54 cases) to overcome the learning curve compared to the NGS team (85 cases) and this difference could be attributed to more experienced personnel and smoother workflows with bioinformatics analyses. The CUSUM showed an overall decrease in the TAT as caseloads increased for both NGS and bioinformatics (**Figure 2C** and **Figure 3C**). For bioinformatics, the improving trend also corresponded with the decrease in overall turnaround time in the EWMA chart for bioinformatics (**Figure 3B**), likely due to the increased efficiency of the bioinformatics team as they gained experience. However, for NGS, there was an overall increasing trend in TAT reflected by the EWMA (**Figure 2B**). This overall increase was attributable to the change of main pathologist operating the NGS equipment (262^nd^ case) and the number of cases analyzed subsequently did not reflect the full learning curve of the NGS team.

An important observation, of a second dip in performance (after 262 cases), was made in the NGS CUSUM chart (**Figure 2C**). This dip was also observed in the EWMA chart (**Figure 2B**), as well as the overall CUSUM and EWMA in **Figure 1**. In this period, two key personnel left the NGS laboratory and there was a handover of work to new manpower. The individual learning curves of transition to new personnel could explain this increase in TAT after 262 cases. The NGS laboratory comprised two to three pathologists involved in tissue quality control, and four to six in the genomics team involved in accessioning, sample preparation, NGS and report preparation. Meanwhile, the bioinformatics laboratory comprised four to six members. While there were changes in personnel in both laboratories, at any given point in time, there was a team focused on IMAC formed by two to three pathologists, two members in the genomics team, and one to two members in the bioinformatics team.

Interestingly, clustering of cases in both NGS (**Figure 2A**) and bioinformatics laboratories (**Figure 3A**) were observed. This habitual clustering of consecutive cases suggests that researchers tended to accumulate samples even after receiving them, before analyzing them as a single batch. Batch processing is a common practice to reduce cost and wastage or reagents, but will consequently impact on TAT if the required number of samples per batch does not materialize within a specified time-frame. Batching samples can also lead to batch-to-batch irregularities in results due to different laboratory conditions, sample degradation and personnel changes (49). Some of these inaccuracies can possibly be reduced by strict standardization of sequencing protocols and training of lab staff.

Within healthcare contexts, the application of control charts has largely been limited to procedural, particularly surgical disciplines (36, 50). The IDEAL Framework for surgical innovation describes five stages of evolution for new surgical therapeutic interventions - Idea, Development, Exploration, Assessment, and Long-term Study (51). This idea can be applied to the field of precision oncology to better the process of finding gene sequences that cause cancers, interpreting these results and finding targeted therapies for these patients. CUSUM and EWMA scrutinizes the NGS and bioinformatics process and assists in mapping the TAT and finding areas of improvement in the evolving process of precision oncology. While the findings of this paper are not necessarily generalizable, it highlights the potential use of statistical process monitoring for precision medicine. As more centres use targeted therapies, analyses should be routinely performed to monitor learning curves and find potential areas of improvement in the process. In doing so, it is important to keep in mind the regional differences which have the potential to influence precision medicine including disparities in the clinical trial landscape and infrastructure, and the sociocultural attitudes towards genetic testing.

### Limitations

This study is limited by the retrospective nature of the data and the fact that the NGS was done in an academic rather than commercial laboratory where changes in a small number of lab personnel and the need to batch cases together can impact on the learning curve of the team and lead to an increase in TAT.

## Conclusions

As NGS and bioinformatics lead precision oncology into the forefront of cancer management, it is vital that the implementation and management of NGS and bioinformatics processes are monitored closely to improve the timeliness of data output and care delivery. By characterizing the TAT using SPC methods, including CUSUM and EWMA, we were able to objectively evaluate how differences in workflows and changes in manpower impacts the performance of precision oncology laboratories. As precision oncology is increasingly adopted, applying SPC prospectively can potentially help spotlight problems early for rectification in NGS and bioinformatics processes.

## Data Availability Statement

The datasets presented in this study can be found in online repositories. The names of the repository/repositories and accession number(s) can be found below: https://www.ncbi.nlm.nih.gov/, https://pubmed.ncbi.nlm.nih.gov/28994108//ijc.31091.

## Author Contributions

Concept and design: NLS, RS, and DT. Acquisition, analysis, or interpretation of data: NLS, SJ, WS, CNg, YC, WL, VH, NK, MG, XL, NSS, XK, ZI, JL, DL, BP, AW, RAS, WY, CC, S-CL, B-CG, RS, and DT. Drafting of the manuscript: SJ, WS, CNg, CH, WL, NLS, NK, MG, and DT. Critical revision of the manuscript for important intellectual content: SJ, WS, CNg, YC, WL, NLS, NK, MG, DT and RS. Statistical analysis: NLS and DT. Study supervision: DT, B-CG, and RS. All authors contributed to the article and approved the submitted version.

## Funding

This investigator-initiated study was funded by the Singapore Ministry of Health’s National Medical Research Council (NMRC) under the Transitional Award Scheme to DT, and its Centre Grant scheme to the National University Cancer Institute, Singapore (NCIS), and the NCIS Yong Siew Yoon (YSY) Cancer Drug Development fellowship to VH. The research is also supported by the National Research Foundation Singapore and the Singapore Ministry of Education under their Research Centres of Excellence (RCE) Initiative. The NMRC had no involvement in the design and conduct of the study; collection, management, analysis and interpretation of the data; preparation, review, or approval of the manuscript; and decision to submit the manuscript for publication.

## Conflict of Interest

DT consults on the advisory board for Astra Zeneca and has received research funding from Karyopharm Therapeutics.

The remaining authors declare that the research was conducted in the absence of any commercial or financial relationships that could be construed as a potential conflict of interest.

## Publisher’s Note

All claims expressed in this article are solely those of the authors and do not necessarily represent those of their affiliated organizations, or those of the publisher, the editors and the reviewers. Any product that may be evaluated in this article, or claim that may be made by its manufacturer, is not guaranteed or endorsed by the publisher.
